# Patients’ experiences of a suppoRted self-manAGeMent pAThway In breast Cancer (PRAGMATIC): quality of life and service use results

**DOI:** 10.1007/s00520-023-08002-z

**Published:** 2023-09-12

**Authors:** V Jenkins, L Matthews, I Solis-Trapala, H Gage, S May, P Williams, D Bloomfield, C Zammit, D Elwell-Sutton, D Betal, J Finlay, K Nicholson, M Kothari, R Santos, E Stewart, S Bell, F McKinna, M Teoh

**Affiliations:** 1 Sussex Health Outcomes Research & Education in Cancer (SHORE-C), Brighton & Sussex Medical School, University of Sussex, Falmer, East Sussex, England UK; 2 School of Medicine, Keele University, University Road, Staffordshire, England UK; 3 Surrey Health Economics Centre/Department of Clinical and Experimental Medicine, Leggett Building, University of Surrey, Guildford, Surrey, England UK; 4 Department of Mathematics, University of Surrey, Guildford, Surrey, England UK; 5 Royal Sussex County Hospital, University Hospitals Sussex NHS Foundation Trust, Brighton, East Sussex, England UK; 6 Surrey & Sussex Cancer Alliance, Royal Surrey County Hospital NHS Foundation Trust, Guildford, Surrey, England UK; 7 Worthing Hospital, University Hospitals Sussex NHS Foundation Trust, Worthing, West Sussex, England UK; 8 Ashford & St Peter’s NHS Foundation Trust, London Road, Ashford, Surrey, England UK

**Keywords:** Breast cancer, Supported self-management, Quality of life, Quality of survivorship, Patient experience, Psychological morbidity

## Abstract

**Purpose:**

To describe trends and explore factors associated with quality of life (QoL) and psychological morbidity and assess breast cancer (BC) health service use over a 12-month period for patients joining the supported self-management (SSM)/patient-initiated follow-up (PIFU) pathway.

**Methods:**

Participants completed questionnaires at baseline, 3, 6, 9 and 12 months that measured QoL (FACT-B, EQ 5D-5L), self-efficacy (GSE), psychological morbidity (GHQ-12), roles and responsibilities (PRRS) and service use (cost diary).

**Results:**

99/110 patients completed all timepoints; 32% (35/110) had received chemotherapy. The chemotherapy group had poorer QoL; FACT-B total score mean differences were 8.53 (95% CI: 3.42 to 13.64), 5.38 (95% CI: 0.17 to 10.58) and 8.00 (95% CI: 2.76 to 13.24) at 6, 9 and 12 months, respectively. The odds of psychological morbidity (GHQ12 >4) were 5.5-fold greater for those treated with chemotherapy. Financial and caring burdens (PRRS) were worse for this group (mean difference in change at 9 months 3.25 (95% CI: 0.42 to 6.07)). GSE and GHQ-12 scores impacted FACT-B total scores, indicating QoL decline for those with high baseline psychological morbidity. Chemotherapy patients or those with high psychological morbidity or were unable to carry out normal activities had the highest service costs. Over the 12 months, 68.2% participants phoned/emailed breast care nurses, and 53.3% visited a hospital breast clinician.

**Conclusion:**

The data suggest that chemotherapy patients and/or those with heightened psychological morbidity might benefit from closer monitoring and/or supportive interventions whilst on the SSM/PIFU pathway. Reduced access due to COVID-19 could have affected service use.

**Supplementary Information:**

The online version contains supplementary material available at 10.1007/s00520-023-08002-z.

## Introduction

The main aims of follow-up after cancer treatment are to monitor for disease recurrence, ensuring patients receive information, support and reassurance to enable them to self-manage and live well beyond their cancer diagnosis and treatment [1]. However, there is little evidence in breast cancer (BC) that intensive follow-up with regular outpatient appointments in secondary care improves the recurrence detection rate or overall survival [2, 3]. A study involving 284 patients in Germany showed that 24% experienced at least a moderate degree of distress prior to their BC follow-up appointments, and although 94% were satisfied with the surveillance regimen, the most important aspect was regular mammography (71%), followed by the clinical consultation (60%) [4].

In the UK National Health Service (NHS), BC follow-up management appears dependent on regional and personal preferences. UK follow-up practice differs from that in other European countries and the USA, where BC survival is often reported as better and regular face-to-face contact is deemed appropriate [5]. The only consensus agreement between the UK, Europe and USA is the continuation of annual or biannual mammography for 10 years [5].

In the second of a series of three papers about cancer survivorship, Jefford and colleagues [6] echo the sentiment that resource constraints mean that current models of follow-up cancer care are unsustainable. The paper summarises evidence from randomised controlled trials on effective pathways and follow-up models, e.g. nurse-led clinics that require modification. One model, supported self-management (SSM) also referred to as patient-initiated follow-up (PIFU), is part of the NHS long-term plan to deliver personalised care to all patients [7]. These stratified follow-up models ensure individual patients are managed on the best follow-up pathway to address their specific needs. Patients are able to self-manage their health, report worrying signs and symptoms as and when necessary to their clinical team, resulting in less time spent attending follow-up clinic appointments. If appropriate, patients continue regular surveillance in the form of tests (e.g. PSA blood test in men with prostate cancer, or mammography for women following breast cancer treatment). Several NHS England Hospital Trusts have engaged in SSM/PIFU and surveillance for those at low risk of recurrence for prostate cancer and colorectal cancer. This has also been introduced for some patients with higher recurrence risks without negatively impacting their quality of life, psychological well-being and lifestyle [7].

In order for patients to be able to manage their own care effectively, they require good preparation, ongoing support and need to possess a moderate to high degree of self-efficacy. According to Bandura’s social cognitive theory, generalised self-efficacy (GSE) beliefs strongly relate to other self-evaluation constructs, including self-esteem, locus of control and neuroticism [8, 9]. Self-efficacy varies depending on the level of task difficulty and certainty of successfully performing that task, relevant when considering stratified follow-up. Bandura’s theory of social construct is complex and incorporates to some extent how familiar one is in dealing with events. Having a diagnosis of breast cancer and self-management of breast cancer is not an event that people can usually prepare well for in advance. However, those with higher levels of self-efficacy may also equate to a better quality of life (QoL). Also, there is evidence to suggest QoL in early-stage BC patients could be impacted by treatments such as chemotherapy and endocrine therapy [10].

Within NHS England, each regional cancer alliance issues its own SSM/PIFU guidelines. These are based on the national guidance where clinical teams decide which patients may benefit based on the cancer type, short- and long-term effects of treatments, other comorbidities and dependency needs. Some of these needs are identified during the Holistic Needs Assessment (HNA) conducted by the nurse specialist to inform the development of a care and support plan. In Surrey and Sussex, several providers have implemented stratified SSM/PIFU in BC where patients continue to have regular surveillance scans or tests (e.g. annual mammograms) but do not attend routine clinic appointments. However, there are few data on long-term health outcomes in SSM/PIFU programmes in cancer, partly because this follow-up approach is fairly new to oncology [11]. There are obvious benefits in terms of clinical time saving, but limited data on patients’ views, impact on psychological wellbeing, QoL and BC-related service use. SSM/PIFU has been introduced recently into the NHS BC world and in PRAGMATIC, we wanted to examine how patients managed their first year, specifically whether or not psychological morbidity or level of confidence (GSE) changed over a 12-month period, and how often they accessed the clinical team.

## Methods

PRAGMATIC was a longitudinal, observational, uncontrolled, mixed-methods study using qualitative (semi-structured interviews) and quantitative (questionnaires) data collection including a health economic evaluation. The quantitative results are presented in this paper.

The aims were to:

- Describe trends in QoL, self-efficacy and psychological morbidity over the first 12 months following hospital-based BC treatment to assess if any changes with respect to baseline at 3-month intervals are maintained longer term.
- Explore factors associated with QoL and psychological morbidity. These include chemotherapy treatment, age, self-efficacy, endocrine treatment and support from a partner.
- Assess BC service use over the 12-month study period, including contacts with hospital breast services, and calculate the associated costs.

### Participants

**Fig. 1:**
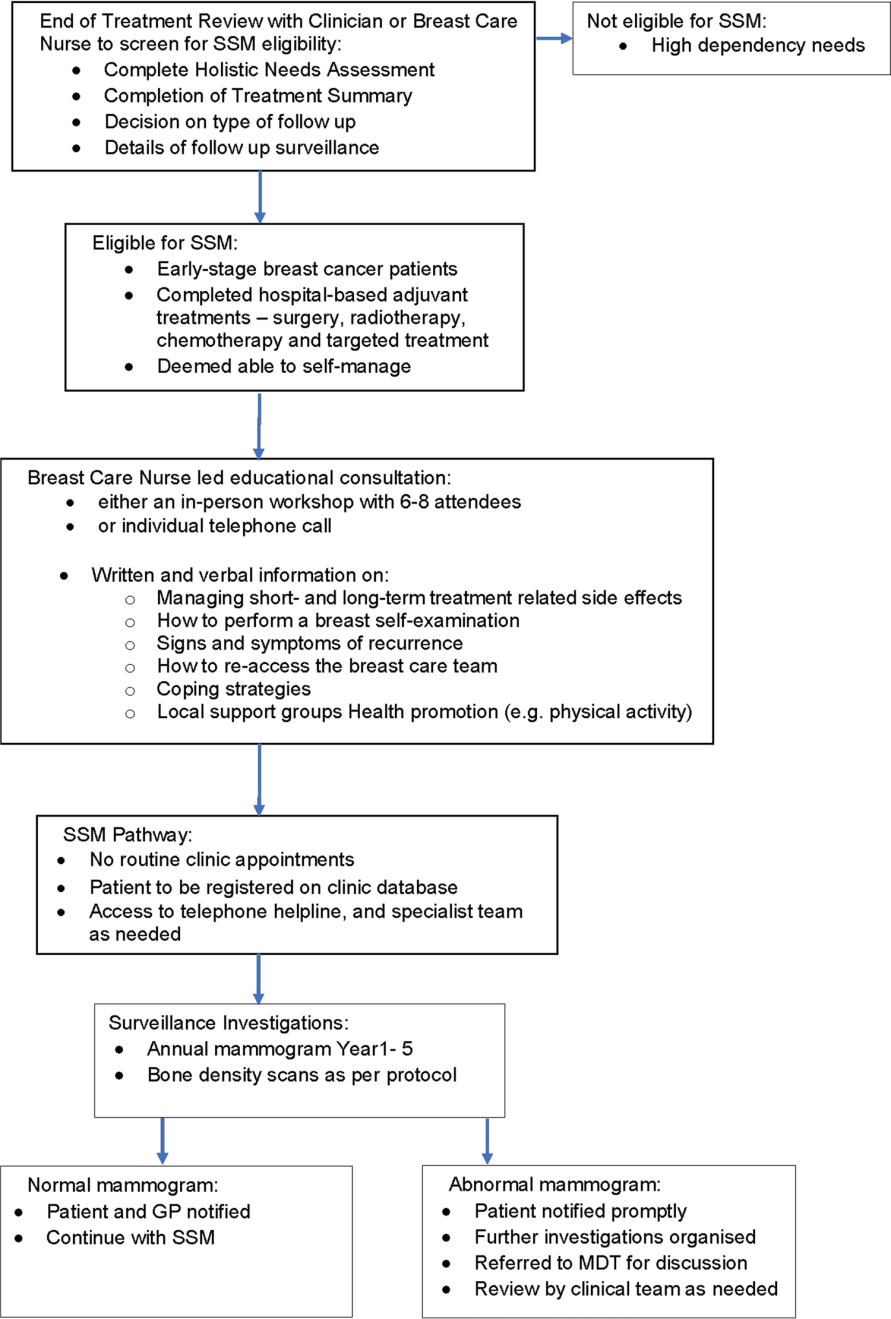
Supported self-management pathway for breast cancer patients

Patients were eligible for PRAGMATIC if they had a diagnosis of early-stage BC, completed adjuvant hospital-based treatment, were considered by their clinical team as being able to access care as needed and thus were suitable for the SSM/PIFU pathway. Figure 1 shows the pathway adopted by the centres.

### Recruitment

The study was introduced to patients at their end of treatment review by the clinical nurse specialists at three hospitals in Sussex and Surrey (Ashford & St Peter’s NHS Foundation Trust, Royal Sussex County Hospital and Western Sussex Hospitals). Recruitment took place between February and November 2020. However, approximately 1 month into the study, a national lockdown was instigated because of the COVID-19 pandemic. This altered the way information about the SSM pathway was provided, with remote consultations replacing face-to-face appointments.

Recruitment was in the ratio of a third post-chemotherapy and two-thirds no chemotherapy, in order to ensure comparability with current UK treatment practice of early-stage BC, where 34% of patients have chemotherapy as part of their primary breast cancer treatment [12]. It was important to examine these two groups (chemotherapy/no chemotherapy) in terms of how they managed the SSM pathway, how often they reconnected with the breast team and highlight if one group required more support than the other.

Recruitment was monitored weekly in order for the centres to approach sufficient numbers of patients in both groups. Those eligible completed an expression of interest form containing contact details, which was sent to researchers at the Sussex Health Outcomes Research and Education in Cancer (SHORE-C) unit, who telephoned them at least 48h later to explain the study in full.

The study received Sponsorship (University of Sussex; 064/JEN/272971) and Health Research Authority Ethics Approval (London-Chelsea REC; 19/LO/1966).

### Data collection

At study entry, we recorded participants’ age, education, living situation and employment status. Clinical details (cancer stage, treatments received) and comorbidities (e.g. arthritis, respiratory conditions) were provided by the hospitals. Consenting participants either completed online (or by paper) questionnaires every 3 months for a period of 1 year, and a subgroup engaged also (at same time-points) in semi-structured interviews (paper under review).

### Quality of life and service use questionnaires

The main objectives were to assess QoL, anxiety/depression and self-efficacy over a 12-month period using the FACT-B (Functional Assessment of Cancer Therapy-Breast) [13], PRRS (Patient Roles & Responsibilities Scale) [14], GHQ-12 (General Health Questionnaire-12 items) [15] and the GSE (General Self-Efficacy Scale) [16].

The EQ-5D-5L (Euro Qol-5D-5L), a five level and five-dimensional generic measure of health-related quality of life, was administered and a EQ-5D-5L health utility index UK score set applied to the health profiles obtained [17]. Breast cancer–related service use was captured using a specially designed questionnaire covering use of primary, community and hospital services, social and voluntary services, help from family and friends and attendance at support groups. Information on private expenditures incurred due to breast cancer were also requested (Appendix A).

The FACT-B produces a total score (sum of all sub-scales) ranging from 0 to 148. A FACT-G score (sum of Physical Wellbeing (PWB) + Social WB +Emotional WB + Functional WB) ranging from 0 to 108 and a FACT-B Trial Outcome Index (TOI). The TOI is the sum of the PWB + FWB + BCS (Breast Cancer Subscale). High scores equate with good QoL.

The PRRS measures the impact that cancer and its treatments have on a patient’s everyday life, such as caring for others, financial and employment responsibilities. The PRRS (responsibilities + family + financial scores) total score ranges from 0 to 64. The jobs and career sub-scale is treated as a standalone score and not included in any total score. Higher scores indicate a better QoL.

The GHQ-12 is a screening tool for identifying minor psychiatric disorders in the general population and within non-psychiatric clinical settings such as general medical outpatients. A score equal to or great than 4 indicates probable psychological morbidity (anxiety combined with depression).

The GSE scale is a10-item self-report unidimensional measure of general self-efficacy. Each item refers to successful coping. Perceived self-efficacy is related to subsequent behaviour and, therefore, is relevant for clinical practice and behaviour change. Items are added to produce a sum score. The range is from 10 to 40 points, with higher scores indicating better self-efficacy.

### Statistical analysis

The proportion of patients whose total FACT-B score either worsened, remained the same or improved at 3, 6, 9 or 12 months with respect to baseline was calculated, using a minimally important difference of 8 points [18]. Additionally, FACT-B total, TOI, BCS, PRRS and GSE scores were modelled longitudinally using linear mixed effects models including a random intercept to account for the correlation amongst the scores collected from the same participant.

Similarly, the probability of psychological morbidity (GHQ-12 equal to or greater than 4) was modelled using a mixed effects logistic regression model with a random intercept.

For the FACT-B total, TOI and BCS scores, two models were fitted. The first model included group (chemotherapy vs no chemotherapy), time and an interaction term for group and time as explanatory variables. The second model added recruitment site, baseline age, GSE score and psychological morbidity to the explanatory variables of the first model. The models for PRRS, GSE and psychological morbidity included group, time and an interaction term for group and time as explanatory variables. Additional models were fitted to see if there was an association between endocrine treatment and psychological morbidity and support from a partner and psychological morbidity. There is some evidence QoL could be affected by endocrine therapy [19], and we wanted to explore if having a partner influenced psychological morbidity.

The models were fitted using maximum likelihood estimation based on all available data. This assumes a missing at random mechanism. Patterns and causes for missingness and dropout from the study were investigated.

Diagnostic plots, including plots of residuals, and Q-Q plots were used to check the model assumptions for each outcome variable. The analyses were carried out using the statistical software R. [20]

### Service use analysis

A costing study was undertaken from the perspectives of the health service and patients. The cost of the intervention was estimated. Service use and breast cancer–related expenditures reported at each of the four assessments were summed for each participant. Data were annualised where the full 12-month data were not available, utilising the mean of a participant’s non-missing scores.

Individual level service use of each item was converted to an annual cost using validated national unit costs [21] (Appendix B). Total costs of all items were summed to produce a total annual cost for each participant. Total costs (logged to account for skew in the data) were modelled using multiple linear regression with forward selection, and with predictors reflecting available patient factors (age group, had chemotherapy or not, cancer grade, tumour stage, EQ-5D-5L mean over 12 months, GHQ-12 mean over 12 months). The cost of the intervention was based on specialist nurse time (see Appendix B) of 1 h (to cover time spent on the phone with the patient, and in preparation and recording notes). Trends in health-related quality of life (EQ-5D-5L) were explored and compared with GHQ-12 scores.

## Results

### Participants

**Fig. 2:**
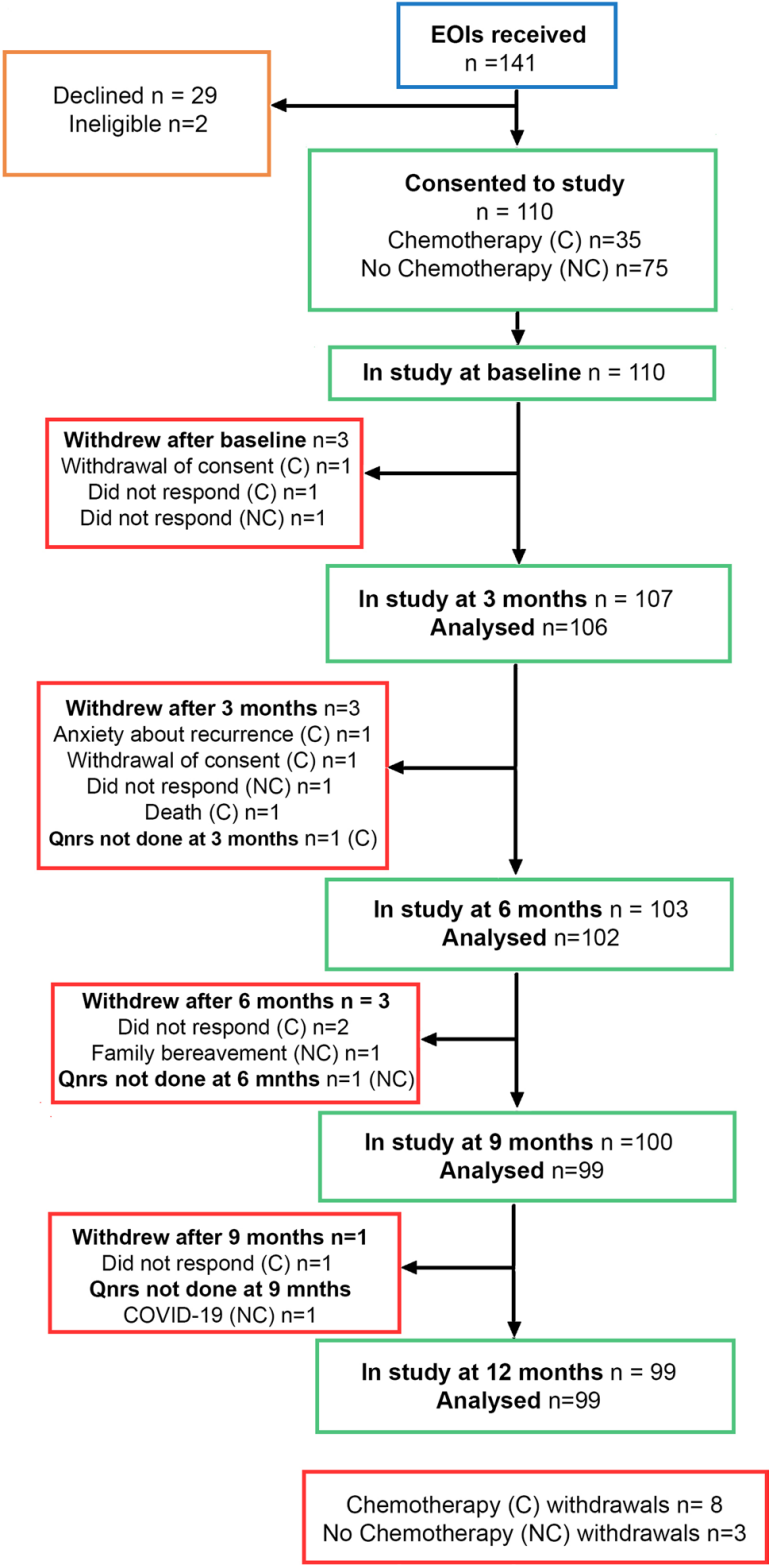
PRAGMATIC consort diagram

110/141 (78%) patients approached (2 men; 108 women) consented to the study, 29/141 (21%) declined and 2 were ineligible. Recruitment and completion of assessments were excellent (*n*=99 at 12 months). One death occurred in the chemotherapy group (secondary to glioblastoma) and 11 participants withdrew during the study (8 chemotherapy; 3 no chemotherapy) (Fig. 2).

Table 1 shows the baseline characteristics; the groups (chemotherapy v no chemotherapy) differed in terms of grade of cancer (grade 3; 66% v 16% respectively), age group (<50years; 37% v 8% and >70years; 6% v 24% respectively) and psychological morbidity (51% v 33%). The majority had at least one comorbidity (73/110; 66%); these were mostly in the older non-chemotherapy patients.

**Table 1:** Baseline characteristics by group

	Chemotherapy (*n*=35)	No chemo (*n*=75)
Sex		
Female	34	74
Male	1	1
Age		
<50yrs	13 (37%)	6 (8%)
50–70yrs	20 (57%)	51(68%)
>70yrs	2 (6%)	18 (24%)
Partner		
Yes	22 (63%)	51(68%)
No	13 (37%)	24(32%)
Education		
Secondary	25 (71%)	52 (69%)
University	10 (29%)	23 (31%)
Employed		
Yes	21 (60%)	32 (43%)
No (inc sick leave)	14 (40%)	43 (57%)
BC grade		
DCIS	0	9 (12%)
I	1(3%)	12 (16%)
II	11 (31%)	40 (53%)
III	23 (66%)	14 (16%)
Surgery*		
Breast conserving	24 (69%)	64 (85%)
Mastectomy	11 (31%)	12 (16%)
Comorbidities		
None	17 (49%)	20 (27%)
1	8 (23%)	23 (31%)
≥2	10 (29%)	32 (43%)
Endocrine therapy		
Yes	26 (74%)	60 (80%)
GHQ-12 >4	18 (51%)	25 (33%)
GSE (mean; sd)	29.8 (4.3)	32.5 (4.5)

*One participant had L BCS and R Mx

### Quality of life

Table 2 shows the proportion of participants where QoL (FACT-B) scores declined, improved or remained the same from baseline. Overall, by 12 months, 51.5% (51/99) remained the same, 24.2% (24/99) improved and 24.2% (24/99) reported a decline in QoL.

**Table 2:** Percentage of participants who improved, declined or had no change from baseline in FACT B total score calculated using a minimally important difference of 8 points

	Chemo				No Chemo			
	*n*	Improve	Decline	No change	*n*	Improve	Decline	No change
3months	32	11 34%	6 19%	15 47%	74	6 8%	15 20%	53 72%
6months	30	12 40%	4 13%	14 47%	72	13 18%	18 25%	41 57%
9months	28	11 39%	6 21%	11 39%	71	12 17%	12 17%	47 66%
12months	27	9 33%	6 22%	12 44%	72	15 21%	18 25%	39 54%

**Fig. 3:**
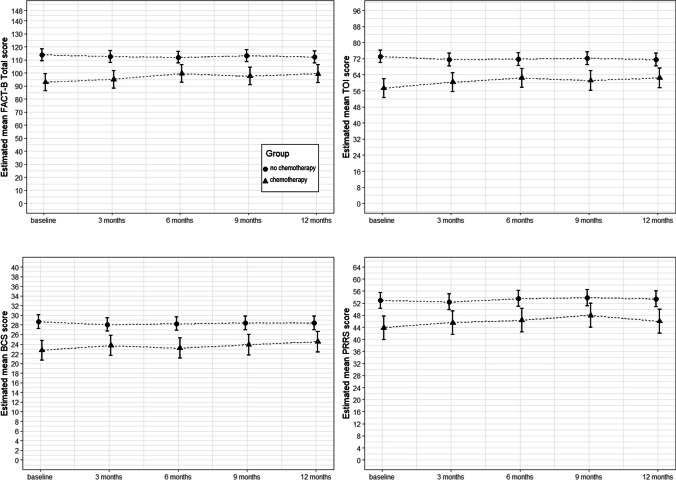
Estimated mean FACT-B total, BCS, TOI and PRRS scores using linear mixed effects models

Results from the linear mixed effects model showed that the chemotherapy group had lower FACT-B total, TOI, BCS and PRRS scores over time compared with those who did not receive chemotherapy (Fig. 3). The mean differences in FACT-B total score changes with respect to baseline between these groups were 8.53 (95% CI: 3.42 to 13.64), 5.38 (0.17 to 10.58) and 8.00 (2.76 to 13.24) at 6, 9 and 12 months respectively (supplementary Table A). These differences were slightly attenuated following adjustment of baseline variables, site of recruitment, age group, GSE score and GHQ-12 score. An increase of one unit on GSE and GHQ baseline scores was associated with mean differences in FACT-B total of 0.88 (0.24 to 1.52) and −21.63 (−27.42 to 15.84) respectively (supplementary Fig. 1a and 1b and supplementary Table B).

The odds of psychological morbidity were estimated to be 5.5-fold greater for a patient treated with chemotherapy, but the 95% CI (1.17 to 25.91) is wide due to the small sample size. Mental health appeared to improve over time for both groups, but this did not reach statistical significance (Supplementary Table C). Slightly smaller effects were obtained from a model including the impact of chemotherapy OR= 4.7 (95% CI: 1.00 to 22.34) and no endocrine therapy OR=6.05 (95% CI: 1.05 to 34.97) together. Having a partner did not affect the odds of experiencing psychological morbidity OR = 1.6 (95% CI: 0.36 to 6.75), and 10 women (4 chemotherapy; 6 no chemotherapy) maintained high levels of psychological morbidity throughout the 12 months of the study.

There was no statistically significant decline from baseline in self-efficacy (GSE scores) for either group (means 31.6 chemotherapy; 31.1 no chemotherapy), but GSE scores and psychological morbidity had a significant effect on the mean FACT-B total score. Mean differences per score unit difference (95% CI) were 0.91 (0.254 to 1.57) and −21.73 (−27.73 to −15.94), respectively (supplementary Table B), showing considerable decline in QoL for participants who had higher levels of psychological morbidity at baseline. Similar results were seen for TOI and BCS, although differences between groups were less marked for the BCS score and there was no effect of GSE score on this subscale.

At baseline 53 (48%), participants said they were employed; 12/53 (23%) stopped work over the course of the study and at 12 months, 5/12 (42%) indicated they intended to return to work. The chemotherapy group had lower PRRS scores over time, but greater improvement occurred at 9 months for this group. Those with a spouse/partner as the main support had better financial resources (mean 17.59, sd 4.97) compared with those with other or no main support (mean 14.6; sd 5.84; mean 13.0, sd 5.43 respectively).

Although the overall completion rate of assessments was very high, we noted that more participants receiving chemotherapy (8/35) compared to those not receiving chemotherapy (3/75) withdrew at different follow-up periods. This difference in dropout patterns may be informative of QoL outcomes; however, this cannot be verified empirically and there were insufficient data to model missingness. Therefore, the results of the longitudinal data analysis which assumed a non-informative dropout mechanism should be interpreted with caution.

### Costs

#### Intervention costs

One hour of a specialist nurse time is approximately £50 (British pounds, 2021)*.*

##### Service use

The most frequently used community service was general practice (54.2% participants contacted their general practitioner at least once and about one-quarter contacted the practice nurses over the 12 months). Other community services were accessed by less than 10% of participants (Table A Health Economics supplementary material). Hospital records were used to estimate contacts with hospital breast care providers because some ambiguities were apparent in the self-reporting of this category of service use. The records indicated that 68.2% had at least one contact with the specialist breast nurses and 53.3% had a clinic visit with a breast specialist doctor (Table B Health Economics supplementary material)**.**

Analysis of annual service use costs indicated considerable variety amongst individuals: mean £350 (excluding a valuation of informal care by family and friends and in-hospital treatment (day cases and overnight stays)) (Table C Health Economics supplementary material). Out of pocket expenditures on BC-related items were reported by one-half of participants, most frequently on clothing or prosthetics and over the counter medications (Table D Health Economics supplementary tables). Regression modelling indicated that the significant drivers of total costs were having had chemotherapy treatment, experiencing psychological morbidity (GHQ-12 ≥4) and reporting being unable to carry out usual activities (EQ-5D-5L usual activity dimension = 5) at one or more assessments during the study period (model parameters Appendix C).

#### Generic health-related quality of life

There was little change in EQ-5D-5L health utility index scores across the 12-month period but, as with the FACT-B results, EQ-5D-5L health utility index scores tended to be lower amongst those who had received chemotherapy treatment (Figs. A & B Health Economics Supplementary material). Mean EQ-5D-5L health utility index scores were also highly negatively correlated with mean GHQ-12 scores, with means calculated using all time points with available scores for each participant (data not shown). Psychological morbidity (GHQ-12 ≥4) was high amongst participants as a whole (39.1%, 43/110, at baseline; 30.3% 30/99 at 12 months).

## Discussion

The PRAGMATIC patient reported outcomes showed that patients treated with chemotherapy had poorer quality of life, heavier financial burdens and greater psychological morbidity when they started the SSM pathway. Also, this study identified a subgroup of patients with heightened psychological morbidity who might benefit from a modified SSM/PIFU model.

In the NHS, most patients complete the Holistic Needs Assessment (HNA) as standard practice at the end of their BC treatment in secondary care. The HNA is used to identify the concerns and needs of individuals, which are discussed at the end of treatment consultation and patients are triaged to support as required [22]. In PRAGMATIC, this information process was impacted by the COVID-19 pandemic, with remote telephone consultations replacing face-to-face or group discussions. It is possible that some concerns were not identified during this time. However, the HNA is not a specific screening tool for psychological morbidity and quality of survivorship, and in PRAGMATIC, anxiety and/or depression significantly impacted QoL more than any other factor. The COVID-19 pandemic undoubtedly may have accentuated feelings of anxiety and depression, as shown in recent literature where cancer survivors experienced heightened levels of psychological distress [23], although in PRAGMATIC, having chemotherapy was a significant factor throughout the follow-up periods in the mixed effects logistic regression model (Supplementary Table C). At least 15% of PRAGMATIC participants reported taking medications and/or attending counselling or classes because of depression, anxiety or stress. In addition, distress, not just anxiety and depression, is increasingly recognised as a factor that negatively impacts on QoL. Exploratory laboratory research suggests stress may potentially compromise cancer control and outcomes by altering the levels of circulating hormones and affecting the immune system [24].

In light of these results, clinical teams may like to consider screening all patients (using GHQ-12), alongside the HNA to identify those who would benefit from extra psychological support. This could include closer monitoring in the form of regular phone calls, nurse-led clinic reviews and/or signposting to relevant psychological support services or other intervention. Recent Cochrane reviews have demonstrated the benefits of physical activity [25], yoga [26] and mindfulness-based stress reduction [27] on improving QoL by reducing anxiety and depression after BC treatment. The economic analysis indicated that psychological morbidity is a significant driver of service use costs, so this mediation would both help the patient and potentially save costs to the NHS.

Consideration could be given also to remotely monitoring patients’ QoL whilst they are self-managing their breast cancer care. One online self-management support package (PROMPT-Care e-PROM) was tested with 221 patients (41.6% breast cancer), and 31 of whom were interviewed about the experience [28]. Patients completed validated patient-reported outcome measures (PROMs) of distress, unmet needs and symptoms every 3 months for 9 months. Those who scored above a pre-determined threshold received an email directing them to relevant online support materials. The findings showed that the resources were perceived as acceptable and useful, with 93% indicating they would reuse them. Although a helpful tool to assist routine follow-up, this method of support is valuable mainly to those with sufficient digital health literacy, who have access to the necessary equipment and good internet connection.

The PRAGMATIC study took place during the COVID-19 pandemic, which affected cancer care worldwide and survivors were expected to take on greater self-management, whilst for some, also dealing with social isolation, financial hardship and loneliness. The pandemic certainly impacted the social and emotional aspects of the PRAGMATIC participants, and those interviewed provided a powerful insight on these issues (paper under review). In contrast, COVID-19 presented few obstacles to recruitment or management of our study, with patients happy to participate. It is challenging, however, with these types of studies to capture the true nature of self-management, as regular completion of PROMs, service use diaries and for some, interviews, may have resulted in a feeling of ongoing support or surveillance. However, the finding of a difference in PROMs between groups makes it unlikely that these participants were displaying the Hawthorne effect, where behaviour is altered because they were part of a study.

PRAGMATIC was limited because there was no control group so it is unknown whether the observed pattern of service use might differ from that of the previous follow-up regimen based on regular hospital outpatient clinic check-ups with doctors. Additionally, the pandemic created a further confounder, potentially reducing the number of service use contacts because face-to-face access was restricted. There was, however, still considerable use of hospital services in the self-management pathway. The open access to specialist breast nurses was taken up by over two thirds of participants, and one-half had at least one clinic visit with a specialist breast doctor.

Consistent with the psychological morbidity identified in PRAGMATIC, the burden of unmet needs (including fear of cancer recurrence) is common especially amongst younger women who receive chemotherapy [29]. This demonstrates that for a small subset of patients, continuing emotional support is needed. However, the benefit of regular routine clinic visits for reassurance is unknown and forms the basis for future research in terms of cost-effectiveness and reduction of health anxiety — particularly fear of cancer recurrence [29].

In conclusion, PRAGMATIC has demonstrated important insights into how patients experience the self-management pathway that has become standard in many regions, so as to accommodate the increasing complexity and numbers of breast cancer patients [30]. Identifying those who could benefit from an alternative model of follow-up with more proactive psychological support could not only improve patients’ quality of life and quality of survival but could also result in potential cost-saving benefits to the NHS. The PRAGMATIC results provide valuable information on approaches to stratification of follow-up for breast cancer and could potentially be practice changing for some providers who have already implemented or are in the process of implementing SSM/PIFU pathways. This is also in line with the aims of the NHS long-term plan for cancer, which recommends that after cancer treatment, every patient should be moved to a follow-up that suits their needs [30].

### Supplementary information

ESM 1 Supplementary Figure Labels (DOCX 23 kb)

ESM 2 Supplementary Tables (DOCX 37 kb)

ESM 3 Supplementary Fig. 1 (JPG 374 kb)

ESM 4 Supplementary Figure (JPG 54 kb)

ESM 5 Supplementary Figure (JPG 69 kb)

## Data Availability

The datasets generated during and/or analysed during the current study are available from the corresponding author on reasonable request.
