# Supplementary material for: Patients’ experiences of a suppoRted self-manAGeMent pAThway In breast Cancer (PRAGMATIC): quality of life and service use results
Source: Support Care Cancer. 2023 Sep 12;31(10):570. doi: 10.1007/s00520-023-08002-z (PMC10497681; doi:10.1007/s00520-023-08002-z)
Supplement: Supplementary file 1 — Supplementary Figure Labels (DOCX 23 kb) [file 520_2023_8002_MOESM1_ESM.docx]

**Supplementary Figure Labels**

**Figure A:** EQ-5D Means with 95% confidence intervals, N=99 completers

**Figure B:** EQ-5D Means with 95% confidence intervals for chemotherapy v no chemotherapy subgroups, N=99 completers

**Figure 1a:** Estimated FACT-B total scores over time using a linear mixed -effects model

**Figure 1b:** Following adjustment of baseline variables
