# Supplementary material for: Patients’ experiences of a suppoRted self-manAGeMent pAThway In breast Cancer (PRAGMATIC): quality of life and service use results
Source: Support Care Cancer. 2023 Sep 12;31(10):570. doi: 10.1007/s00520-023-08002-z (PMC10497681; doi:10.1007/s00520-023-08002-z)
Supplement: Supplementary file 2 — Supplementary Tables (DOCX 37 kb) [file 520_2023_8002_MOESM2_ESM.docx]

**Supplementary Tables**

**Supplementary Table A:** Linear mixed-effects model for FACT-B total score

| *Explanatory variables Beta estimate* | | *95% CI* | *p-value* |
| --- | --- | --- | --- |
| Group [no chemo] | Reference | | |
| Group [chemo] | -20.98 | -29.03 to-12.94 | <0.001 |
| Time [baseline] | Reference | | |
| Time [3 months] | -1.41 | -4.18 to 1.35 | 0.316 |
| Time [6 months] | -1.81 | -4.60 to 0.98 | 0.203 |
| Time [9 months] | -0.64 | -3.44 to2.17 | 0.655 |
| Time [12 months] | -1.53 | -4.32 to 1.26 | 0.283 |
| Group [chemo] * Time [3 months] | 3.60 | -1.41 to8.61 | 0.159 |
| Group [chemo] * Time [6 months] | 8.53 | 3.42 to13.64 | 0.001 |
| Group [chemo] * Time [9 months] | 5.38 | 0.17 to 10.58 | 0.043 |
| Group [chemo] * Time [12 months] | 8.00 | 2.76 to13.24 | 0.003 |

**Variance components**

Within-individual variance 73.37

Between-individual variance 326.86

Intraclass correlation 0.82

Number of individuals 110

Number of observations 516

**Supplementary Table B:** Linear mixed-effects model for FACT-B total score adjusting for baseline variables

| *Explanatory variables Beta estimate* | | *95% CI* | *p-value* | |  |
| --- | --- | --- | --- | --- | --- |
| Site [Ashford and St Peter's Hospital] | Reference | | | |  |
| Site [Royal Sussex County Hospital] | 2.35 | -3.99 to 8.68 | | 0.467 | |
| Site [Worthing Hospital] | 3.52 | -2.52 to 9.55 | | 0.253 | |
| Age group [<50] | Reference | | | |  |
| Age group [50-60] | -1.68 | -9.61 to 6.26 | 0.678 | |  |
| Age group [61-70] | 3.00 | -5.14 to11.13 | 0.470 | |  |
| Age group [>70] | 3.53 | -5.71 to12.76 | 0.454 | |  |
| Baseline GSE score | 0.91 | 0.25 to1.57 | 0.007 | |  |
| Baseline GHQ score | -21.73 | -27.52 to-15.94 | <0.001 | |  |
| Group [no chemo] | Reference | | | |  |
| Group [chemo] | -13.60 | -20.39 to -6.82 | <0.001 | |  |
| Time [baseline] | Reference | | | |  |
| Time [3 months] | -1.40 | -4.17 to 1.36 | 0.320 | |  |
| Time [6 months] | -1.78 | -4.57 to1.01 | 0.210 | |  |
| Time [9 months] | -0.63 | -3.43 to2.17 | 0.659 | |  |
| Time [12 months] | -1.51 | -4.30 to1.28 | 0.289 | |  |
| Group [chemo] * Time [3 months] | 3.64 | -1.37 to 8.64 | 0.154 | |  |
| Group [chemo] * Time [6 months] | 8.25 | 3.14 to13.35 | 0.002 | |  |
| Group [chemo] * Time [9 months] | 5.17 | -0.02 to10.37 | 0.051 | |  |
| Group [chemo] * Time [12 months] | 7.77 | 2.53 to13.00 | 0.004 | |  |

**Variance components**

Within-individual variance 73.43

Between-individual variance 160.10

Intraclass correlation 0.69

Number of individuals 110

Number of observations 516

**Supplementary Table C:** Mixed-effects logistic regression model for the probability of a GHQ-12 score equal to or greater than 4 (psychological morbidity)

| *Explanatory variables* | *Odds ratio* | *95% CI* | *p-value* |
| --- | --- | --- | --- |
| Group [no chemo] | Reference | | |
| Group [chemo] | 5.51 | 1.17to 25.91 | 0.031 |
| Time [baseline] | Reference | | |
| Time [3 months] | 0.60 | 0.26 to 1.38 | 0.232 |
| Time [6 months] | 0.51 | 0.22 to1.19 | 0.117 |
| Time [9 months] | 0.52 | 0.22 to 1.22 | 0.132 |
| Time [12 months] | 0.48 | 0.20 to1.13 | 0.093 |
| **Variance components** | | | |
| Within-individual variance | 3.29 | | |
| Between-individual variance | 9.13 | | |
| Intraclass correlation | 0.74 | | |
| Number of individuals | 110 | | |
| Number of observations | 516 | | |

**Health Economic Supplementary Tables**

**Table A: Annualised primary and community service use (breast cancer related) – number of visits/ consultations, N=107, self-report questionnaire data**

| **Area** | **Question** | **Item** | **Ever used in 12m Yes, n** | **Ever used in 12m**  **Yes, %** | **Mean** | **Maximum** |
| --- | --- | --- | --- | --- | --- | --- |
| GP | 1 | Visited GP | 39 | 36.4 | 0.77 | 9 |
|  | 2 | Advice from GP by phone or email | 50 | 46.7 | 1.57 | 18 |
|  | 3 | Visited at home by GP | 1 | 0.9 | 0.02 | 2 |
|  | *1+2+3* | *Any GP contact* | *58* | *54.2* |  |  |
|  | 4 | Seen Nurse at GP surgery | 27 | 25.2 | 0.52 | 7 |
| Home contact  (not hospital nurse) | 5 | Advice from Nurse by phone or email | 23 | 21.5 | 0.52 | 8 |
|  | 6 | Visited at home by Nurse | 1 | 0.9 | 0.06 | 6 |
|  | 7 | Visited at home by Care Worker | 1 | 0.9 | 0.24 | 26 |
| Additional treatments | 8 | Advice from Physiotherapist | 11 | 10.3 | 0.43 | 23 |
|  | 9 | Advice from Dietician | 6 | 5.6 | 0.09 | 4 |
|  | 10  For anxiety, depression, stress | Medications* | 15 | 14.0 | NA | NA |
|  |  | Counselling | 6 | 5.6 | 1.51 | 52 |
|  |  | Hypnotherapy | 1 | 0.9 | 0.24 | 26 |
|  |  | Support group | 1 | 0.9 | 0.22 | 24 |
|  |  | Other | 5 | 4.7 | 1.85 | 100 |
|  | 11  Complementary  Therapy | Acupuncture | 6 | 5.6 | 1.08 | 26 |
|  |  | Osteopathy | 3 | 2.8 | 0.64 | 48 |
|  |  | Massage (includes Reike and scar treatment therapy) | 3 | 2.8 | 0.52 | 40 |
|  |  | Chiropractor / | 3 | 2.8 | 0.28 | 14 |
|  |  | Other | 2 | 1.9 | 0.11 | 8 |
| Additional help / support | 19 | Attended a breast cancer support group | 9 | 8.4 | 0.42 | 18 |
|  | 20 | At home, from family and friends | 10 | 9.3 | 3.35 | 138.67 |
|  | 21 | Other service / professional not included above | 36 | 33.6 | 0.98 | 22 |

* Some respondents didn’t provide the frequency of taking medications so summary statistics could not be computed / are not available

**Table B: Annualised hospital use (breast cancer related), from hospital records, N=107**

| **Area** | **Question** | **Item** | **From hospital records** | | | |
| --- | --- | --- | --- | --- | --- | --- |
|  |  |  | **Ever used in 12m Yes, n** | **Ever used in 12m Yes, %** | **Mean** | **Maximum** |
| Hospital use | 12 | Visited specialist breast doctor (consultant) in hospital clinic | 57 | 53.3 | 1.25 | 12 |
|  | 13 | Visited breast nurse at hospital | 41 | 38.3 | 0.58 | 5 |
|  | 14 | Spoke to hospital breast nurse by phone | 73 | 68.2 | 1.36 | 12 |
|  | *13+14* | *Any breast nurse contact* | *73* | *68.2* |  |  |
|  | 15 | Exchanged emails with hospital breast nurse | 3 | 2.8 | 0.06 | 4 |
|  | 16 | Attended A&E | 0 | 0 | 0 | 0 |
|  | 17 | Had day hospital treatment | 12 | 11.2 | 0.24 | 4 |
|  | 18 | Stayed overnight (number of nights) | 1 | 0.9 | 0.09 | 1 |

**Table C: Yearly costs of service use (British pounds 2021), medications and personal expenditures excluded; hospital costs based on hospital records**

|  | **N** | **Mean** | **SD** | **Lower 95% CI** | **Upper 95% CI** | **Maximum** |
| --- | --- | --- | --- | --- | --- | --- |
| Family and friends support | 107 | 56.98 | 308.40 | -2.33 | 116.30 | 2357 |
| GP, community, family/friends support, hospital excluding day cases and inpatient stay | 106 | 413.91 | 660.31 | 286.74 | 541.08 | 3340 |
| Hospital day cases and inpatient stay | 106 | 213.84 | 624.84 | 93.50 | 334.18 | 3360 |
| **Grand total cost [GP, community, family/friends support, all hospital]** | 106 | 627.75 | 889.43 | 456.45 | 799.04 | 3919 |

**Table D: Annualised private expenditures, £ (breast cancer related), N=107, self-report questionnaire data (question 22)**

| **Area** | **Question**  **22** | **Cost Item** | **Ever incurred cost in 12m Yes, n** | **Ever incurred cost in 12m Yes, %** | **Mean** | **Maximum** |
| --- | --- | --- | --- | --- | --- | --- |
| Private expenditures on breast cancer related items | a | Travel to treatment visits (taxi, bus, parking etc) | 24 | 22.4 | 22.12 | 1400 |
|  | b | Prescription medications | 3 | 2.8 | 1.52 |  |
|  | c | Other medications purchased (over the counter) | 32 | 29.9 | 25.20 | 400 |
|  | d | Special garments / prosthetics | 25 | 23.4 | 40.94 | 840 |
|  | e | Other expenditure* | 16 | 15.0 | 28.46 | 1200 |
|  |  | Total | 53 | 49.5 | 118.24 | 3000* |

* One outlier reporting other expenditure of £3000 was excluded treated as missing) as the data could not be verified; one other participant happened to have a maximum total cost of £3000.
