# Supplementary figures and images for: Patients’ experiences of a suppoRted self-manAGeMent pAThway In breast Cancer (PRAGMATIC): quality of life and service use results
Source: Support Care Cancer. 2023 Sep 12;31(10):570. doi: 10.1007/s00520-023-08002-z (PMC10497681; doi:10.1007/s00520-023-08002-z)

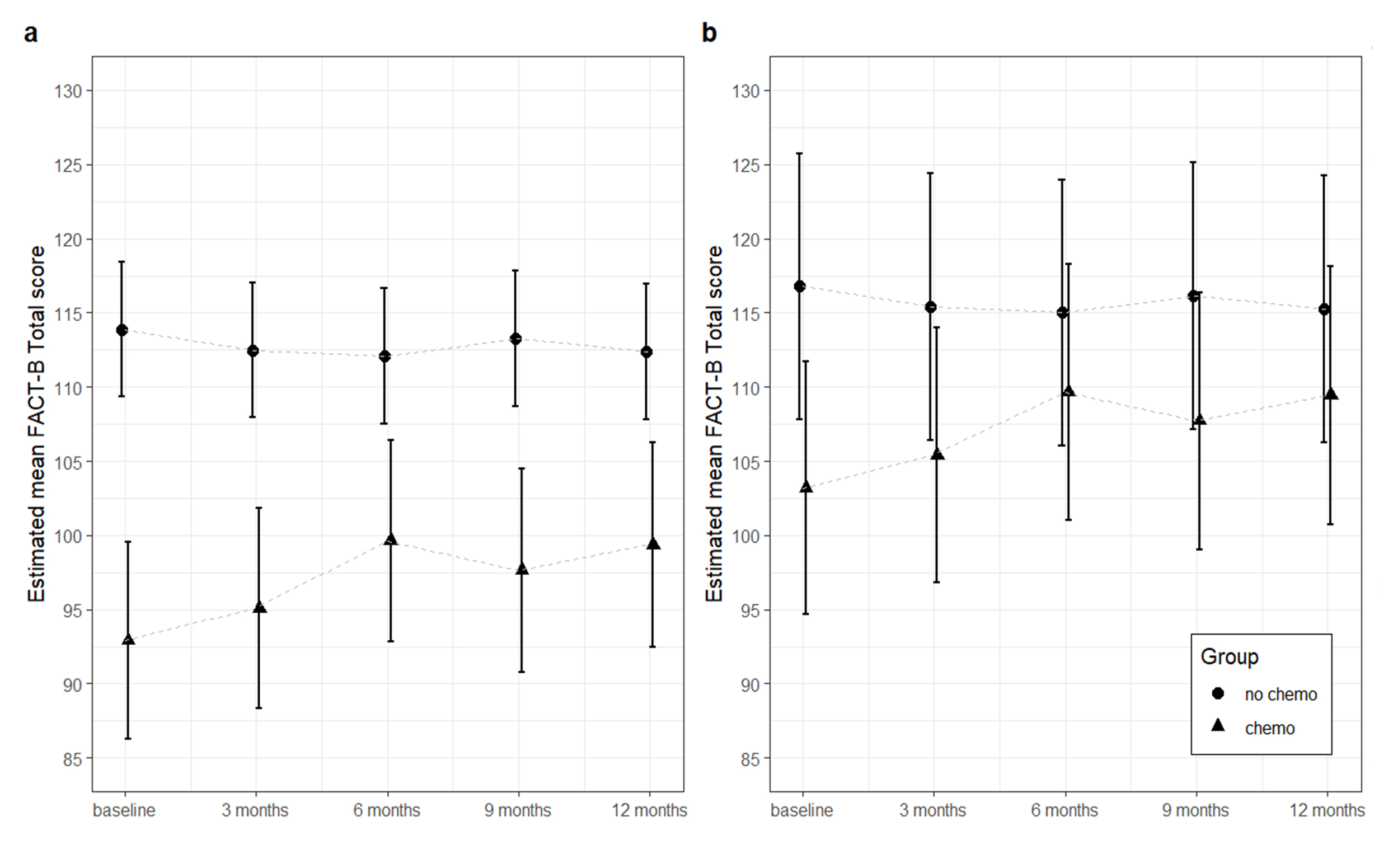

Supplement: Supplementary file 3 — Supplementary Fig. 1 (JPG 374 kb) [file 520_2023_8002_MOESM3_ESM.jpg]

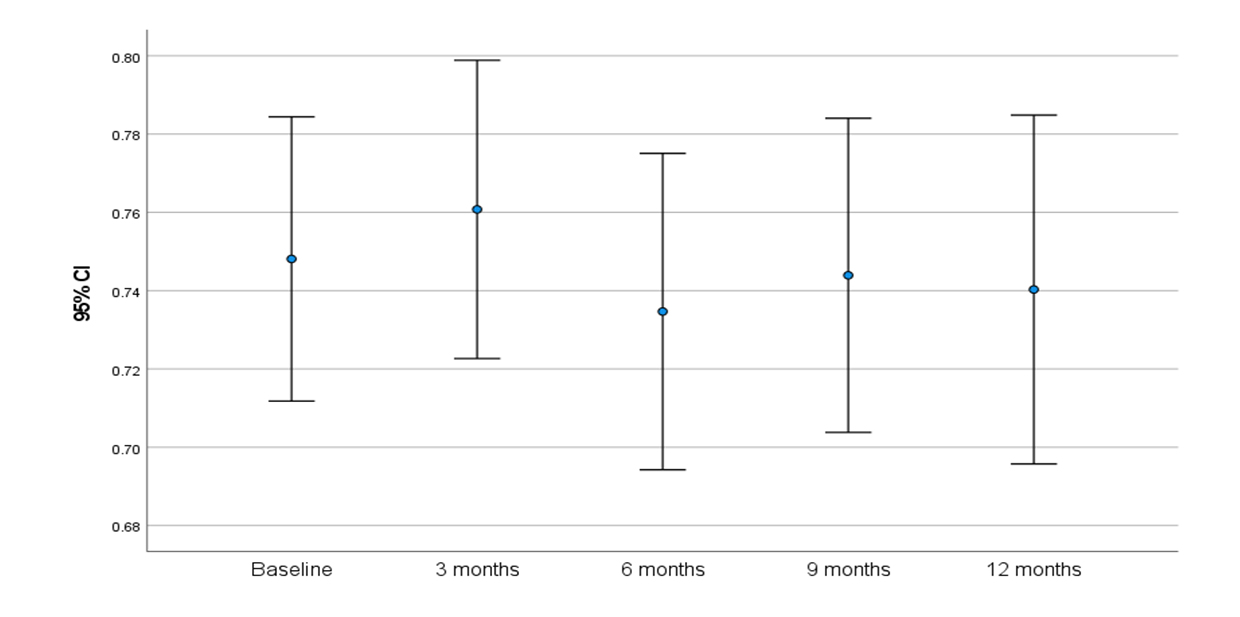

Supplement: Supplementary file 4 — Supplementary Figure (JPG 54 kb) [file 520_2023_8002_MOESM4_ESM.jpg]

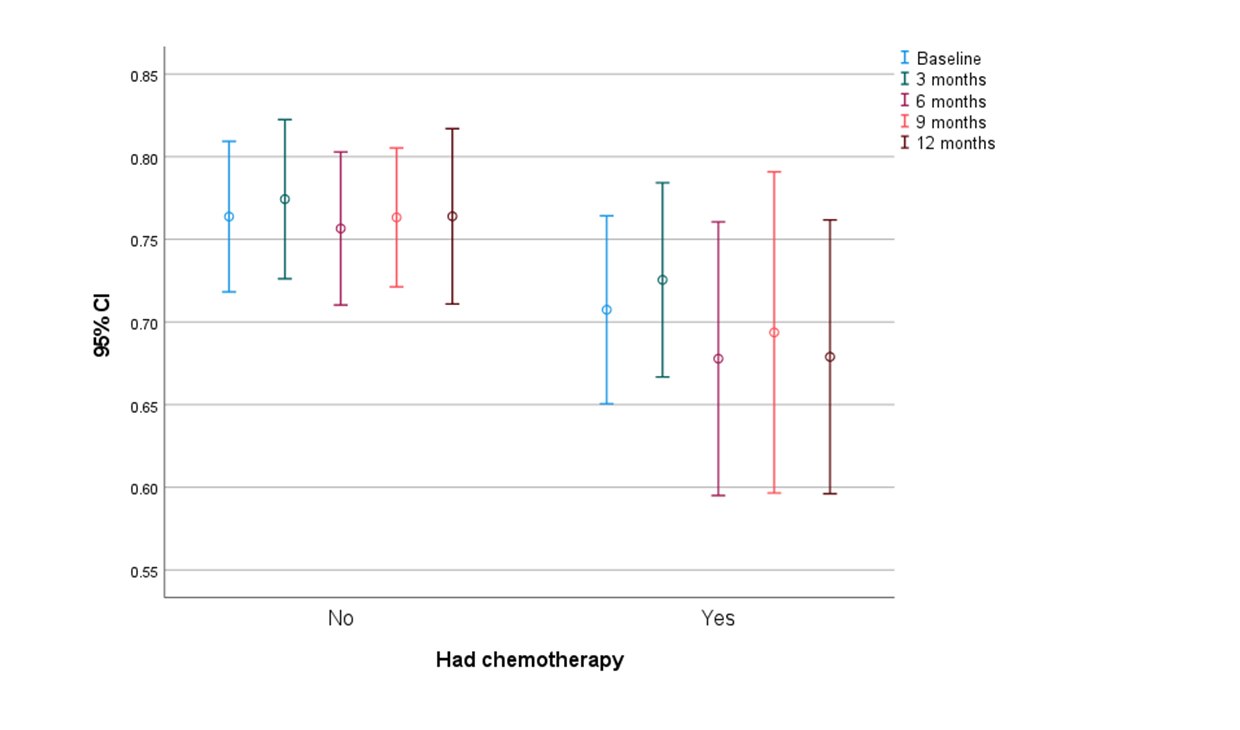

Supplement: Supplementary file 5 — Supplementary Figure (JPG 69 kb) [file 520_2023_8002_MOESM5_ESM.jpg]
